# Microstructural Improvement of Eutectic Al + Mg_2_Si Phases on Al–Zn–Si–Mg Cast Alloy with TiB_2_ Particles Additions

**DOI:** 10.3390/ma14112902

**Published:** 2021-05-28

**Authors:** Byungjoo Kim, Jihoon Hwang, Yongho Park, Youngcheol Lee

**Affiliations:** 1 Energy Component & Material R&BD Group, Korea Institute of Industrial Technology, Busan 46938, Korea; kbj@kitech.re.kr; 2 Department of Materials Science and Engineering, Pusan National University, Busan 46241, Korea; 3 Materials & Production Engineering Research Institute, LG Electronics, Pyeongtaek 17709, Korea; lightalloy.hwang@lge.com

**Keywords:** aluminum alloy, phase modification, intermetallic compound, eutectic Al + Mg_2_Si phases

## Abstract

In this study, the effects of adding TiB_2_ particles to eutectic Al + Mg_2_Si phases in aluminum alloys were analyzed. The eutectic Al + Mg_2_Si phases were modified effectively when a large amount of TiB_2_ was added, and changes in the shape, size, and distribution of the eutectic Al + Mg_2_Si phases were confirmed using a polarizing microscope and FE-SEM. The crystal structure of the TiB_2_ particles and Mg_2_Si phases were analyzed using HR-TEM, and the analysis confirmed that the TiB_2_ particles can act as heterogeneous nucleation sites. This paper intends to clarify the principle of phase modification of the eutectic Al + Mg_2_Si phases by TiB_2_ particles and proposes a new mechanism to improve Mg_2_Si phase modification when TiB_2_ particles are added.

## 1. Introduction

Mg_2_Si is an intermetallic compound (intermetallics) with excellent hardness (4.5 × 10^9^ N/m^2^), modulus of elasticity (120 GPa), and low density (1.99 g/cm^3^) [1,2]. The shape, size, and distribution of Mg_2_Si phases have a significant effect on its mechanical properties. In Al–Si–Mg alloy systems, Mg_2_Si phases are crystallized according to the ratio of Si and Mg composition. In as-cast conditions, Mg_2_Si phases grow in a coarse dendritic shape [3,4,5,6]. Coarse dendritic Mg_2_Si phases lead to nonhomogeneous stress concentration and reduce the mechanical properties of Al alloys. Therefore, improving the morphologies of the Mg_2_Si phases is a decisive test for Al–Mg_2_Si alloys. In previous studies, it has been reported that Mg_2_Si phases in Al-based alloys are improved by the application of microstructure treatment processes such as hot extrusion [7] and modification heat treatment [8]. In addition, Mg_2_Si phase improvement can be achieved with the addition of P [5,9], Na [10], TiB_2_ [9,11,12], and Ca/Sb [13,14]. However, most of these studies have focused on the improvement of the primary Mg_2_Si phase. There are only a few studies that have been conducted on eutectic Al + Mg_2_Si phases. The shape of the eutectic Al + Mg_2_Si phases has a great influence on the mechanical properties as well as on the primary Mg_2_Si phase. In general, only a few hundred ppm additions of alloying elements or agents are expected to yield enough effects for the modification or phase improvement of aluminum alloys [15,16,17]. However, in the case of Mg_2_Si some studies have reported that a relatively large amount of TiB_2_ particles (approximately 5 wt.% [12] and 5 vol.% [18]) were required to effectively improve the eutectic Al + Mg_2_Si phases of Al–Mg_2_Si alloys. In our previous work, TiB_2_ particles (approximately 1 wt.% Ti) effectively improved the eutectic Al + Mg_2_Si phases of Al-based alloys [11].

There are various types of eutectic (Al + Mg_2_Si) colonies. Variations in these colonies depend on the contents of Si and Mg such as lamellar, flake-likes, rod-likes, and irregular lamella [19]. In studies of the improvement of eutectic Al + Mg_2_Si phases, the modified eutectic Al + Mg_2_Si phases were still eutectic colony types [10,20,21]. Interestingly, the eutectic (Al + Mg_2_Si) colony was changed to a divorced eutectic colony by the addition of Al–5Ti–1B master alloys. However, there is still insufficient evidence to provide an explanation of the relationship between the relatively large amount of TiB_2_ particles and the eutectic (Al + Mg_2_Si) colony improvements. The purpose of this study is to investigate the relationship between TiB_2_ and eutectic Al + Mg_2_Si phases in an Al–Zn–Si–Mg–Cu cast alloy. In addition, the modified mechanism of the eutectic Al + Mg_2_Si phases with TiB_2_ particles was also confirmed.

## 2. Materials and Methods

**Figure 1:**
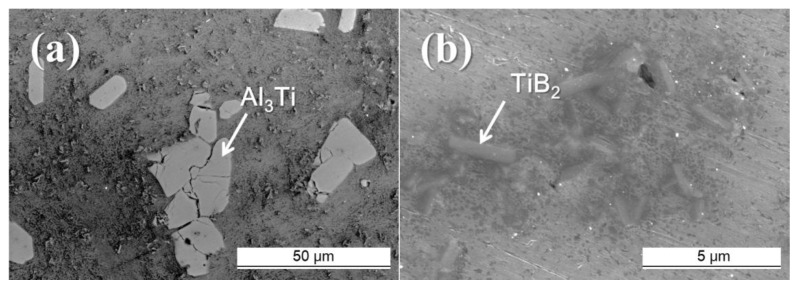
SEM image of the Al–5Ti–1B master alloy rod: (**a**) low magnification and (**b**) high magnification.

The Al–8Zn–6Si–4Mg–2Cu–xTi (x = 0, 0.1, 0.5, and 1) casting alloy was manufactured using gravity casting. A high-frequency induction melting furnace was used for melting at 680 °C ± 5 °C. The alloy composition (all compositions quoted in this work are given in wt.%) was aligned using commercial Al (99.97%), Zn (99.9%), Mg (99.8%), and Cu (99.9%) ingots; pure crystalline Si (99.9%); and the Al–5Ti–1B master alloy rod. Figure 1 shows the Al–5Ti–1B master alloy as observed through a field emission scanning electron microscope (FE-SEM). As evidenced in Figure 1, the microscope shows the presence of both TiB_2_ and Al_3_Ti particles in the Al–5Ti–1B master alloy. The Al–5Ti–1B master alloy was added after all other elements were dissolved entirely. The manufactured alloys were analyzed by optical emission spectrometer (SPECTRO MAXx, SPECTRO, Kleve, Germany), and the composition details of this analysis are shown in Table 1. The specimen for metallographic observation was obtained from the same location as the sample manufactured from the molten metal cylinder mold (32 Ø × 70 mm, FC25 cast iron) preheated to 250 °C. The microstructure was observed using the FE-SEM (S-4800, HITACHI, Tokyo, Japan) polarizing microscope and 200 kV FE-transmission electron microscopy (Talos F200X G2 TEM, Thermo Fisher Scientific, Waltham, MA, USA). A fluoboric acid–distilled water solution was used as an etchant for electrolytic polishing (Lectropol-5, Struers, Copenhagen, Denmark). Average grain size measurements were conducted according to ASTM E1382 standards.

## 3. Results

### 3.1. Eutectic Al + Mg_2_Si Phase Modification and Microstructure Change by Al–5Ti–1B Master Alloy Addition

**Figure 2:**
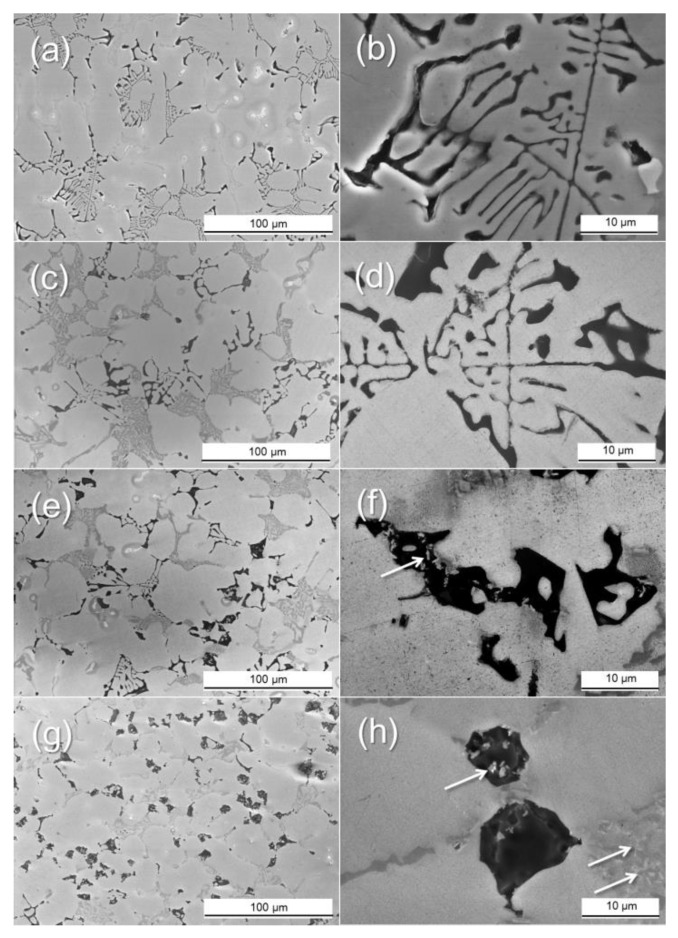
SEM images of the eutectic Al + Mg_2_Si phases of the Al–8Zn–6Si–4Mg–2Cu alloy with different Ti amounts: (**a**,**b**) without Ti; (**c**,**d**) at 0.1% of Ti; (**e**,**f**) at 0.5% of Ti; and (**g**,**h**) at 1% of Ti. Here, the white arrows indicate the TiB_2_ particles.

**Figure 3:**
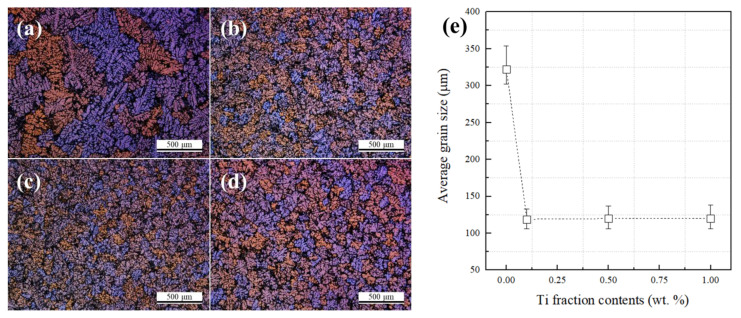
Polarized microscope images of the Al–8Zn–6Si–4Mg–2Cu alloy with different amounts of Ti: (**a**) without Ti; (**b**) at 0.1% of Ti; (**c**) at 0.5% of Ti; (**d**) at 1% of Ti; and (**e**) the relationship between average grain size and Ti content.

**Figure 4:**
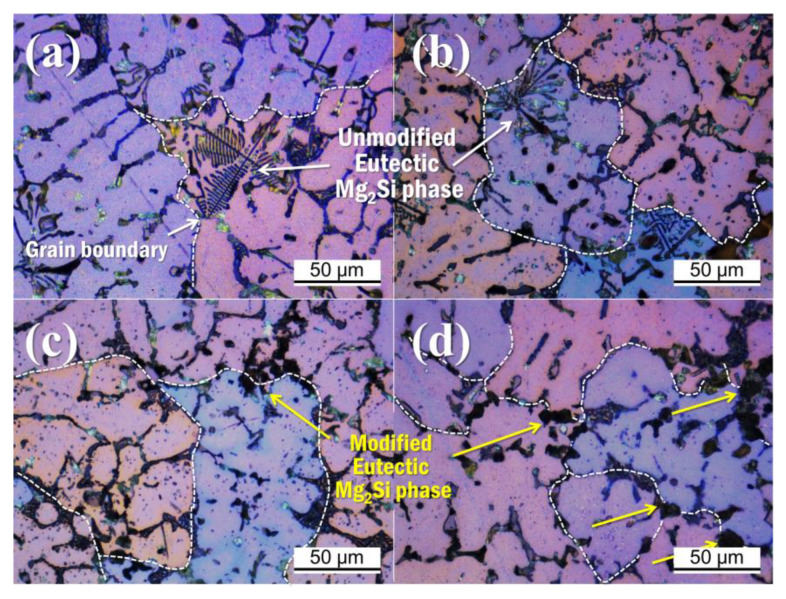
Polarized microscope images (high magnification) of the Al–8Zn–6Si–4Mg–2Cu alloys with different amounts of Ti: (**a**) without Ti; (**b**) at 0.1% of Ti; (**c**) at 0.5% of Ti; and (**d**) at 1% of Ti. Here, the white dotted line represents the grain boundaries. The white and yellow arrows represent unmodified and modified eutectic Al + Mg_2_Si phases, respectively.

Figure 2 shows the change of the eutectic Al + Mg_2_Si phases with different Ti amounts in the Al–8Zn–6Si–4Mg–2Cu alloys. The Chinese script-type eutectic Al + Mg_2_Si phases can be seen in the microstructure of the base alloy. When 0.1% of Ti was added, the shape of the eutectic Al + Mg_2_Si phases remained a Chinese script type. When 0.5% of Ti was added, both the Chinese script type and polygonal structure of the eutectic Al + Mg_2_Si phases were observed (Figure 2e). When 1% of Ti was added, the eutectic Al + Mg_2_Si phases’ morphology changed into a fine polygonal structure (Figure 2b,d). In the same figure, TiB_2_ particles (indicated by white arrows) can be observed in both the inner and outer parts of the eutectic Al + Mg_2_Si phases (Figure 2h). Figure 3 and Figure 4 show the polarizing microscope image of the Al–8Zn–6Si–4Mg–2Cu alloy with different amounts of Ti addition. Here, the different colors represent different grains. The average grain size was measured according to ASTM E1382 standards using IMT I-solution DT software (ver. 11.2, IMT i–Solution, Rochester, NY, United States). When 0.1% of Ti was added to the base alloy, the average grain size decreased from 322 to 120 μm. However, no further grain refining was observed when the Ti addition was increased to 1%. While the unmodified eutectic Al + Mg_2_Si phases were observed at the edges of the Al grains (shown in Figure 4a,b), the modified eutectic Al + Mg_2_Si phases were observed at the grain boundaries (Figure 4b,c).

### 3.2. TEM/EDS and HR-TEM Results of Modified Eutectic Al + Mg_2_Si Phases

**Figure 5:**
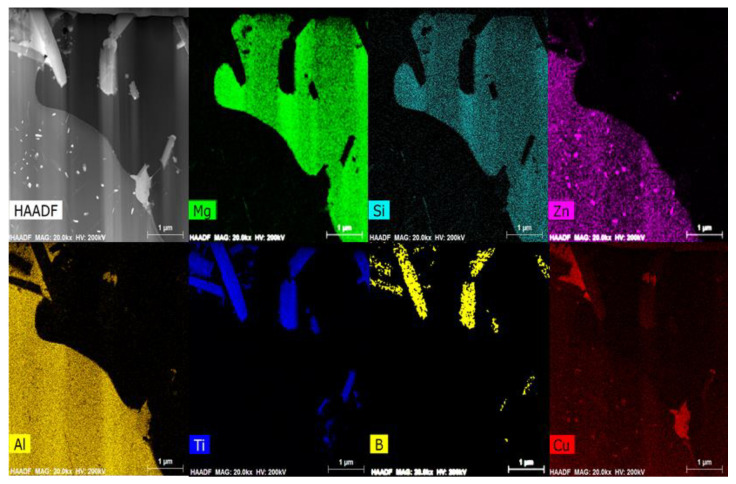
TEM and EDS analysis data of modified eutectic Al + Mg_2_Si phases in the Al–8Zn–6Si–4Mg–2Cu–1Ti alloy.

**Figure 6:**
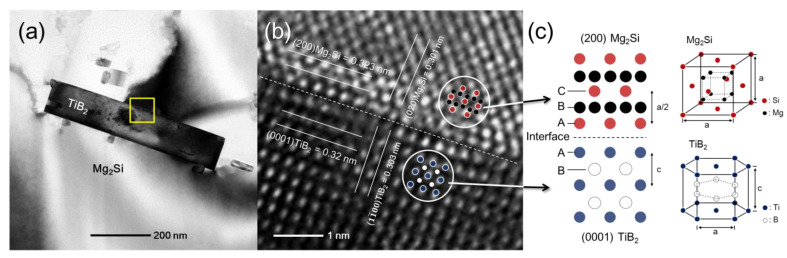
TEM micrographs of the modified eutectic Al + Mg_2_Si phases in the Al–8Zn–6Si–4Mg–2Cu–1Ti alloys: (**a**) the bright field TEM image of the modified eutectic Al + Mg_2_Si phases with TiB_2_ particles; (**b**) is the corresponding high-resolution TEM image of the yellow box in (**a**); and (**c**) the illustration of stacking order of (200)_Mg₂Si_ plane and (0001)_TiB₂_ plane.

Figure 5 shows a cross section of the modified eutectic Al + Mg_2_Si phases analyses by TEM/EDS (Transmission Electron Microscope/Energy Dispersive X-ray Spectroscopy). The Al, Mg, Si, Ti, and B elements were detected. The TiB_2_ particles (the area where Ti and B signals overlap) are observed inside the modified eutectic Al + Mg_2_Si phases. Figure 6 shows the TEM micrographs of the modified eutectic Al + Mg_2_Si phases in the Al–8Zn–6Si–4Mg–2Cu–1Ti alloys. Figure 6a shows the bright field TEM image of the modified eutectic Al + Mg_2_Si phases. Figure 6b is a high-resolution TEM image of the yellow box in Figure 6a, which shows the interface between Mg_2_Si and TiB_2_.

## 4. Discussion

### 4.1. Nucleation Sites of the Eutectic Al + Mg_2_Si Phases on the TiB_2_ Particles

Figure 4 shows that, as the Ti amount increased from 0% to 1%, more of the eutectic Al + Mg_2_Si phases was modified. The Al–5Ti–1B master alloy usually contains a 1.94 volume fraction of TiB_2_ particles. Although this volume fraction is low, the Al–5Ti–1B master alloy contains a large amount of TiB_2_ particles (Figure 1) because TiB_2_ particles can be as small as 1–4 μm (in Figure 1b). The Al–8Zn–6Si–4Mg–2Cu–1Ti alloy contains a 0.39 volume fraction of TiB_2_ particles. TiB_2_ particles were also observed around the modified eutectic Al + Mg_2_Si phases (Figure 2h). These results indicate that the TiB_2_ particles acted as nucleation sites for the eutectic Al + Mg_2_Si phases. However, the eutectic Al + Mg_2_Si phases were unmodified by the addition of 0.1% (0.04 vol.% of TiB_2_ particles) and 0.5% of Ti (0.19 vol.% of TiB_2_ particles). In these Al–8Zn–6Si–4Mg–xTi (x = 0.1 and 0.5) alloys, the amount of TiB_2_ particles was insufficient to change the shape of the eutectic Al + Mg_2_Si phases. When 1% of Ti was added, there was a sufficient number of TiB_2_ particles to act as nucleation sites for the eutectic Al + Mg_2_Si phases. For this reason, most of the eutectic Al + Mg_2_Si phases were modified.

Figure 5 shows the TEM/EDS data for the modified eutectic Al + Mg_2_Si phases and the TiB_2_ particles. The TiB_2_ particles were observed inside the improved eutectic Al + Mg_2_Si phases and were also contained in the Al–5Ti–1B master alloy used for the Al–8Zn–6Si–4Mg–2Cu–xTi alloys (in Figure 1). Since the melting temperature of TiB_2_ particles is 3225 °C [22], they did not melt easily at the temperature required to melt the Al alloy. The TiB_2_ particles could therefore act as heterogeneous nucleation sites for eutectic Al + Mg_2_Si phases during the solidification process.

Figure 6a shows the TEM images of the modified eutectic Al + Mg_2_Si phases. TiB_2_ particles were observed in the modified eutectic Al + Mg_2_Si phases. Figure 6b is a high-resolution TEM image of the yellow box in Figure 6a. The crystallographic structure of the TiB_2_ particle and the eutectic Al + Mg_2_Si phases are clearly observed. The upper section shows the eutectic Al + Mg_2_Si phases, and the lower section shows the TiB_2_ particle. The crystal orientations of both phases were measured by HR-TEM. The lattice plane spacing of the lower section crystal (α) is 0.32 nm, which is in agreement with the (0001) plane of the TiB_2_ crystal structure [3]. The lattice plane spacing of the upper section crystal is 0.323 nm, which is in agreement with the (200) plane of the Mg_2_Si crystal structure [9]. The stacking order of the (200)_Mg₂Si_ plane and the (0001)_TiB₂_ plane is illustrated in Figure 6c. The stacking order of the (200)_Mg₂Si_ plane is ABC…, where the stacking pattern of Si atoms (ACA…) is observed in Figure 6b. The stacking order of (0001)_TiB₂_ plane is ABA…, where the stacking pattern of Ti atoms (AA…) is observed. The atomic patterns of Mg and B are not observed due to differences in zone axis. The dotted line in Figure 6b shows an interface of Mg_2_Si and TiB_2_ that is clearly well bonded. The crystal plane of (200)_Mg₂Si_ and (0001)_TiB₂_ possesses a low misfit (approximately 4.64%), as calculated by the Turnbull–Vonnegut equation [9]. Therefore, the TiB_2_ particle clearly acted as the heterogeneous nucleation site for the eutectic Al + Mg_2_Si phases.

### 4.2. Eutectic Al + Mg_2_Si Phase Modification and Microstructure Change by TiB_2_ Particle Additions

**Figure 7:**
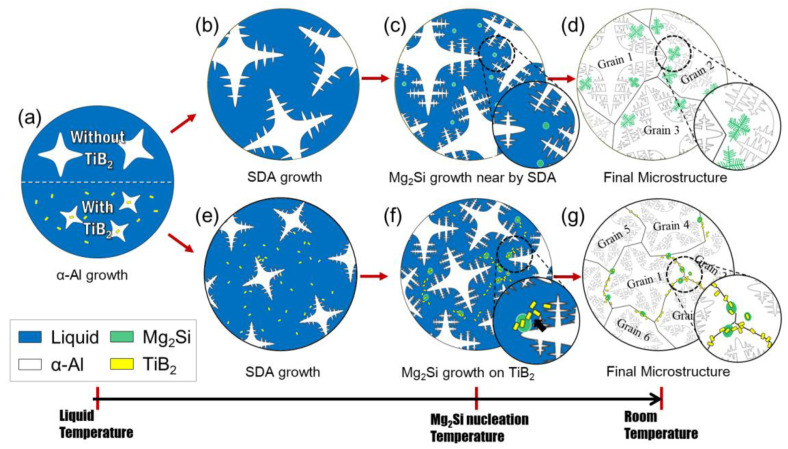
Schematic presentation of the solidification processes of eutectic Al + Mg_2_Si phases in aluminum alloys: (**a**)→(**b**)→(**c**)→(**d**) without TiB_2_ particles; (**a**)→(**e**)→(**f**)→(**g**) with TiB_2_ particles.

As shown in Figure 2a,b, the morphology of the eutectic Al + Mg_2_Si phases is in the form of the Chinese script shape. Observations under a polarizing microscope showed the unmodified eutectic Al + Mg_2_Si phases at the inner edges of the Al grains (in Figure 4a,b). Since the eutectic Al + Mg_2_Si phases are located inside of the Al grains, it can be concluded that there was eutectic (Al + Mg_2_Si) growth from the primary Al grains during the solidification process. Figure 7a–d illustrates the microstructural evolution during solidification of the unmodified eutectic Al + Mg_2_Si phases. Figure 7a demonstrates the formation of the nucleated α-Al by the Al solidification reaction inside the molten metal. Figure 7b shows the growth of the secondary dendrite arm (SDA) due to the growth and coarsening of the α-Al phase. The solute element (Mg and Si) diffusion is a result of the Al phase growth. The solute element rich zone surrounds the growing solid Al phase. The formation of the Mg_2_Si nuclei emerged as a result of constitutional super-cooling from the SDA of α-Al phase. While the eutectic Al + Mg_2_Si phases grew near the SDA, the α-Al phase also grew. This indicates that there was contact between both phases during solidification. Separation of the Al elements occurred around the growing eutectic Al + Mg_2_Si phases. The edges of eutectics Mg_2_Si have a low potential for liquid/α-Al interface. Therefore, the α-Al phase easily engulfed the eutectic Al + Mg_2_Si phases. Consequently, the eutectic Al + Mg_2_Si phases solidified at the edges of the Al grains, as shown in Figure 4a,b and Figure 7d.

In the hypoeutectic composition of Al–Mg_2_Si alloys, the eutectic Al + Mg_2_Si phases solidify after the α-Al phase growth. When the eutectic reaction begins, the eutectic Al + Mg_2_Si phases are crystallized in the remaining liquid phase. Therefore, effective improvement of the eutectic Al + Mg_2_Si phases occurs only when the TiB_2_ particles remain in the liquid phase. However, since the TiB_2_ particles have an excellent crystallographic match with the Al phase, most of the TiB_2_ particles acted as heterogeneous sites for the α-Al phase [23]. Therefore, a small amount of the TiB_2_ particles was easily encased in the Al grains. This is why the eutectic Al + Mg_2_Si phases were not effectively improved when 0.1% and 0.5% of Ti were added to the Al–8Zn–6Si–4Mg–2Cu alloys. However, once the TiB_2_ particles were sufficiently aggregated in the molten metal, the agglutinated TiB_2_ particles had a high potential for the growing liquid (molten metal)/solid (α-Al) interface. Therefore, during the α-Al phase growth, the agglutinated TiB_2_ particles were easily pushed out by the Al grains [24,25,26]. When the eutectic reaction began, a large amount of the TiB_2_ particles existed around the SDA of the Al phase.

Figure 7a,e–g demonstrates the solidification mechanism of the eutectic Al + Mg_2_Si phases when sufficient TiB_2_ particles were added. Figure 7a,e shows the nucleation and growth of α-Al. Here, the TiB_2_ particles aggregated in the liquid phase. These particles were pushed out by the Al grains and now exist around the SDA. When the eutectic reaction began, the TiB_2_ particles acted as heterogeneous nucleation sites for the eutectic Al + Mg_2_Si phases (in Figure 7f). The eutectics Mg_2_Si were simultaneously crystallized in the TiB_2_ particles’ agglomeration region. The growing eutectic Al + Mg_2_Si phases interfered with each other and prevented coarse growth. The TiB_2_ particles were located around the grown eutectics Mg_2_Si. The α-Al phase also grew during the nucleation of the eutectic Al + Mg_2_Si phases. Unlike the unmodified eutectic Al + Mg_2_Si phases, the modified eutectic Al + Mg_2_Si phases and aggregated TiB_2_ particles had a high potential for a liquid/solid interface. They were easily pushed out to the grain boundaries (in Figure 7g). Therefore, in the final microstructure, the modified eutectic Al + Mg_2_Si phases and the TiB_2_ particles are located at the Al grain boundaries (in Figure 4d).

Another important point of discussion is the effect of grain refinement by the addition of TiB_2_ on the shape of the eutectic Al + Mg_2_Si phases. The Al–5Ti–1B master alloy is a well-known Al grain refiner [27,28]. The average grain size was greatly reduced from 322 to 122 μm when 0.1% Ti was added to the Al–8Zn–6Si–4Mg–2Cu alloy. However, when either 0.5 or 1 wt.% Ti was added, the grains were not further refined. As seen in Figure 4a,b, the shape and location of eutectic Al + Mg_2_Si phases did not change due to grain refinement. Therefore, grain refinement does not affect the modification of the eutectic Al + Mg_2_Si phases.

### 4.3. Effect of the Addition of TiB_2_ Particles to Al–8Zn–6Si–4Mg–2Cu Alloys

**Figure 8:**
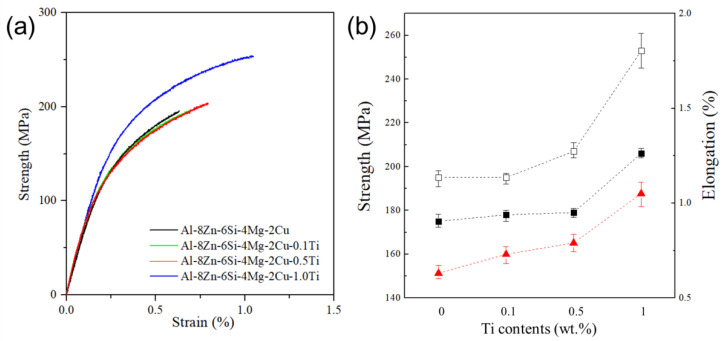
(**a**) Representative strain–stress curve of the Al–8Zn–6Si–4Mg–2Cu–xTi (x = 0, 0.1, 0.5, and 1) alloys; (**b**) yield strength, elongation, and ultimate tensile strength of the Al–8Zn–6Si–4Mg–2Cu–xTi alloys [11].

The effect of the addition of TiB_2_ particles on the mechanical properties of the Al–8Zn–6Si–4Mg–2Cu alloys is given in Figure 8 [11]. A tensile test of the Al–8Zn–6Si–4Mg–2Cu–xTi (x = 0, 0.1, 0.5, and 1) alloy was conducted according to ASTM E8 standards. Elongations of individual alloys used strain values at the time of tensile failure. The value of yield strength was confirmed by the “0.2 off-set” method. The morphology of eutectic Al + Mg_2_Si phases significantly affected its mechanical properties. Yield strength, ultimate tensile strength, and elongation were increased by the addition of TiB_2_ particles (Figure 8b). While the Ti content increased to 0.5%, the tensile behavior of the Al–8Zn–6.4Si–4Mg–2Cu–xTi (x = 0, 0.1, and 0.5) alloys did not change significantly (Figure 8a). However, when 1% Ti was added, the mechanical properties increased significantly. In the Al–8Zn–6.4Si–4Mg–2Cu–xTi (x = 0, 0.1, and 0.5) alloys, eutectic Al + Mg_2_Si phases were coarse, irregular, and located at the end of the aluminum grains. The tips of the unmodified Mg_2_Si were close to the aluminum grains and led to nonhomogeneous stress concentrations. Therefore, the unmodified Mg_2_Si phase caused micro-cracks and intergranular fracture. This provides an explanation for why the mechanical properties did not increase significantly. However, modified eutectic Al + Mg_2_Si phases caused homogeneous stress concentration. Since modified eutectic Al + Mg_2_Si phases were located at the boundary of the aluminum grains, it prevented the propagation of intergranular fracture. Therefore, the mechanical properties of the Al–8Zn–6Si–4Mg–2Cu–1Ti alloy increased significantly.

## 5. Conclusions

The eutectic Al + Mg_2_Si phases of the Al–8Zn–6Si–4Mg–2Cu alloy was effectively modified when 1% of Ti was added. The morphologies of the eutectic structures changed from a coarse Chinese script to a fine polygonal shape. TiB_2_ particles were observed in the modified eutectics Mg_2_Si. The crystal structures of both phases were analyzed by HR-TEM to confirm that the TiB_2_ particles were excellent heterogeneous nucleation particles for Mg_2_Si.

The modified Mg_2_Si phase moved from the inside of the grain to the grain boundary, and TiB_2_ particle clustering around the improved phase was observed. The TiB_2_ particles agglomerated in the molten Al alloy were easily pushed by the growing primary Al. These particles could remain in the residual molten metal until the eutectic Al + Mg_2_Si phases’ solidification temperature was reached. However, individual TiB_2_ particles were easily surrounded by the growing primary aluminum matrix and did not have a significant effect on the improvement of the eutectic Al + Mg_2_Si phases.

## Figures and Tables

**Table 1:** Chemical composition of different Al–5Ti–1B master alloy additions to the Al–8Zn–6Si–4Mg–2Cu–xTi alloys. All values are expressed in weight percent.

Alloy Components	Zn	Si	Mg	Cu	Ti	Al
Al–8Zn–6Si–4Mg–2Cu (Base)	7.96	5.94	4.02	1.97	<0.003	bal.
Base + 0.1% of Ti	7.98	5.98	4.01	1.96	0.13	bal.
Base + 0.5% of Ti	8.01	5.94	3.97	1.95	0.49	bal.
Base + 1% of Ti	7.99	6.01	4.05	2.01	1.02	bal.

## Data Availability

The raw data required to reproduce these findings are available for download from http://dx.doi.org/10.17632/23zxhtgmht.1 (accessed on 21 October 2020).

## References

[1] Li J., An Q., Wu S., Li F., Lü S., Guo W. (2019). Relationship of Mg_2_Si morphology with Mg_2_Si content and its effect on properties of in-situ Mg_2_Si/Al–Cu composites. J. Alloys Compd..

[2] Lu L., Lai M.O., Hoe M.L. (1998). Formation of nanocrystalline Mg_2_Si and Mg_2_Si dispersion strengthened Mg-Al alloy by mechanical alloying. Nanostructured Mater..

[3] Li C., Wu Y.Y., Li H., Liu X.F. (2011). Morphological evolution and growth mechanism of primary Mg_2_Si phase in Al-Mg_2_Si alloys. Acta Mater..

[4] Yu H.C., Wang H.Y., Chen L., Liu F., Wang C., Jiang Q.C. (2015). Heterogeneous nucleation of Mg2Si on CaSb_2_ nucleus in Al-Mg-Si alloys. CrystEngComm.

[5] Li C., Liu X., Wu Y. (2008). Refinement and modification performance of Al-P master alloy on primary Mg_2_Si in Al-Mg-Si alloys. J. Alloys Compd..

[6] Qin Q., Li W. (2016). The Formation and Characterization of the Primary Mg_2_Si Dendritic Phase in Hypereutectic Al-Mg_2_Si Alloys. Mater. Trans..

[7] Ghandvar H., Idris M.H., Ahmad N. (2018). Effect of hot extrusion on microstructural evolution and tensile properties of Al-15%Mg_2_Si-xGd in-situ composites. J. Alloys Compd..

[8] Nasiri N., Emamy M., Malekan A., Norouzi M.H. (2012). Microstructure and tensile properties of cast Al-15%Mg_2_Si composite: Effects of phosphorous addition and heat treatment. Mater. Sci. Eng. A.

[9] Li C., Liu X., Zhang G. (2008). Heterogeneous nucleating role of TiB_2_ or AlP/TiB_2_ coupled compounds on primary Mg_2_Si in Al-Mg-Si alloys. Mater. Sci. Eng. A.

[10] Emamy M., Khorshidi R., Raouf A.H. (2011). The influence of pure Na on the microstructure and tensile properties of Al-Mg_2_Si metal matrix composite. Mater. Sci. Eng. A.

[11] Kim B.J., Jung S.S., Hwang J.H., Park Y.H., Lee Y.C. (2019). Effect of Eutectic Mg_2_Si Phase Modification on the Mechanical Properties of Al-8Zn-6Si-4Mg-2Cu Cast Alloy. Metals.

[12] Du R., Yuan D., Li F., Zhang D., Wu S., Lü S. (2019). Effect of in-situ TiB_2_ particles on microstructure and mechanical properties of Mg_2_Si/Al composites. J. Alloys Compd..

[13] Yu H.C., Wang H.Y., Chen L., Zha M., Wang C., Li C., Jiang Q.C. (2017). Spheroidization of primary Mg_2_Si in Al-20Mg2Si-4.5Cu alloy modified with Ca and Sb during T6 heat treatment process. Mater. Sci. Eng. A.

[14] Yu H.C. (2019). Crystallization of primary Mg_2_Si in Al-20Mg_2_Si alloy with various molar ratios of Ca/Sb. J. Alloys Compd..

[15] Lin Y.C., Luo S.C., Huang J., Yin L.X., Jiang X.Y. (2018). Effects of solution treatment on microstructures and micro-hardness of a Sr-modified Al-Si-Mg alloy. Mater. Sci. Eng. A.

[16] Kang H.S., Yoon W.Y., Kim K.H., Kim M.H., Yoon Y.P., Cho I.S. (2007). Effective parameter for the selection of modifying agent for Al-Si alloy. Mater. Sci. Eng. A.

[17] Lu L., Nogita K., Dahle A.K. (2005). Combining Sr and Na additions in hypoeutectic Al-Si foundry alloys. Mater. Sci. Eng. A.

[18] Gao Q., Wu S., Lü S., Duan X., Zhong Z. (2015). Preparation of in-situ TiB_2_ and Mg_2_Si hybrid particulates reinforced Al-matrix composites. J. Alloys Compd..

[19] Zhu X., Yang H., Dong X., Ji S. (2019). The effects of varying Mg and Si levels on the microstructural inhomogeneity and eutectic Mg_2_Si morphology in die-cast Al–Mg–Si alloys. J. Mater. Sci..

[20] Farahany S., Ghandvar H., Nordin N.A., Ourdjini A., Idris M.H. (2016). Effect of Primary and Eutectic Mg_2_Si Crystal Modifications on the Mechanical Properties and Sliding Wear Behaviour of an Al–20Mg2Si–2Cu–xBi Composite. J. Mater. Sci. Technol..

[21] Li C., Wu Y., Li H., Wu Y., Liu X. (2010). Effect of Ni on eutectic structural evolution in hypereutectic Al-Mg_2_Si cast alloys. Mater. Sci. Eng. A.

[22] Munro R.G. (2000). Material properties of titanium diboride. J. Res. Natl. Inst. Stand. Technol..

[23] Dong X., Youssef H., Zhang Y., Wang S., Ji S. (2019). High performance Al/TiB_2_ composites fabricated by nanoparticle reinforcement and cutting-edge super vacuum assisted die casting process. Compos. Part B Eng..

[24] Youssef Y.M., Dashwood R.J., Lee P.D. (2005). Effect of clustering on particle pushing and solidification behaviour in TiB_2_ reinforced aluminium PMMCs. Compos. Part A Appl. Sci. Manuf..

[25] Wang X., Brydson R., Jha A., Ellis J. (1999). Microstructural analysis of Al alloys dispersed with TiB_2_ particulate for MMC applications. J. Microsc..

[26] Schaffer P.L., Miller D.N., Dahle A.K. (2007). Crystallography of engulfed and pushed TiB_2_ particles in aluminium. Scr. Mater..

[27] Pattnaik A.B., Das S., Jha B.B., Prasanth N. (2015). Effect of Al-5Ti-1B grain refiner on the microstructure, mechanical properties and acoustic emission characteristics of Al5052 aluminium alloy. J. Mater. Res. Technol..

[28] Amerioon A., Emamy M., Ashuri G. (2015). Investigation the Effect of Al-5Ti-1B Grain Refiner and T6 Heat Treatment on Tensile Properties of Al-8%Mg. Procedia Mater. Sci..

